# Cerebral Oxygenation and Metabolism After Hypoxia-Ischemia

**DOI:** 10.3389/fped.2022.925951

**Published:** 2022-07-12

**Authors:** Simerdeep K. Dhillon, Eleanor R. Gunn, Benjamin A. Lear, Victoria J. King, Christopher A. Lear, Guido Wassink, Joanne O. Davidson, Laura Bennet, Alistair J. Gunn

**Affiliations:** Fetal Physiology and Neuroscience Group, Department of Physiology, The University of Auckland, Auckland, New Zealand

**Keywords:** cerebral blood flow, hypoxia-ischemia brain, biomarkers, fetal sheep, neonatal encephalopathy, monitoring

## Abstract

Perinatal hypoxia-ischemia (HI) is still a significant contributor to mortality and adverse neurodevelopmental outcomes in term and preterm infants. HI brain injury evolves over hours to days, and involves complex interactions between the endogenous protective and pathological processes. Understanding the timing of evolution of injury is vital to guide treatment. Post-HI recovery is associated with a typical neurophysiological profile, with stereotypic changes in cerebral perfusion and oxygenation. After the initial recovery, there is a delayed, prolonged reduction in cerebral perfusion related to metabolic suppression, followed by secondary deterioration with hyperperfusion and increased cerebral oxygenation, associated with altered neurovascular coupling and impaired cerebral autoregulation. These changes in cerebral perfusion are associated with the stages of evolution of injury and injury severity. Further, iatrogenic factors can also affect cerebral oxygenation during the early period of deranged metabolism, and improving clinical management may improve neuroprotection. We will review recent evidence that changes in cerebral oxygenation and metabolism after HI may be useful biomarkers of prognosis.

## Introduction

Perinatal hypoxia-ischemia (HI) is a major cause of brain injury and contributes to 0.6 million neonatal deaths globally every year and approximately 1.3 million intrapartum stillbirths (1). In high income countries, moderate to severe hypoxic-ischemic encephalopathy (HIE) occurs in ∼1–4 infants per 1,000 live births at term, leading to death and severe disabilities such as cognitive and neuromotor impairments, including cerebral palsy (2). Brain injury in preterm infants is multifactorial, but rates of HIE in preterm infants are higher than at term (3). Preterm birth is associated with about one-third of all cases of cerebral palsy (4), and high rates of motor deficits, cognitive and language delay, and behavioral problems (5). Therapeutic hypothermia is standard care for term infants with moderate-severe HIE to reduce death and disability. However, the neuroprotection is partial, as many infants still survive with disability despite treatment with therapeutic hypothermia, and its safety for preterm infants has not been established (6).

One of the central challenges is to identify infants at risk of developing brain injury and poor outcomes, and in particular, term infants with mild HIE and preterm infants show subtle neurological signs (7, 8). Past clinical studies have primarily focused on neonates with moderate to severe HIE. There is emerging evidence that infants with mild HIE are at increased risk of adverse neurodevelopmental outcomes (9). As a result, there has been therapeutic drift, with many centers offering therapeutic hypothermia for infants with mild HIE despite limited systematic evidence for benefit or harm (10). Ongoing clinical trials (NCT04621279 and NCT04176471) are assessing effectiveness of therapeutic hypothermia for mild HIE.

Finding ways to reliably identify the stage of injury is critical. For effective neuroprotection, therapeutic hypothermia needs to be started within 6 h from birth, corresponding with the transient recovery of mitochondrial function and metabolism during the so called “latent” phase (6). This latent phase typically lasts for about 6–8 h after moderate to severe HI. This is followed by a phase of secondary deterioration of oxidative metabolism, cytotoxic edema and ultimately bulk cell death lasting for ∼72 h (11). Even after this time, there is considerable evidence for tertiary evolution of ongoing injury and dysmaturation, but also ongoing repair processes that may last for weeks to months (12, 13). These phases of evolving injury after HI are associated with characteristic neurophysiological and cerebral perfusion changes. We will discuss the temporal profile of these changes during recovery after HI, and review recent evidence that changes in cerebral oxygenation and metabolism may have value as prognostic markers.

### The Latent Phase: Post-hypoxic-ischemic Neural Suppression and Delayed Hypoperfusion

**FIGURE 1:**
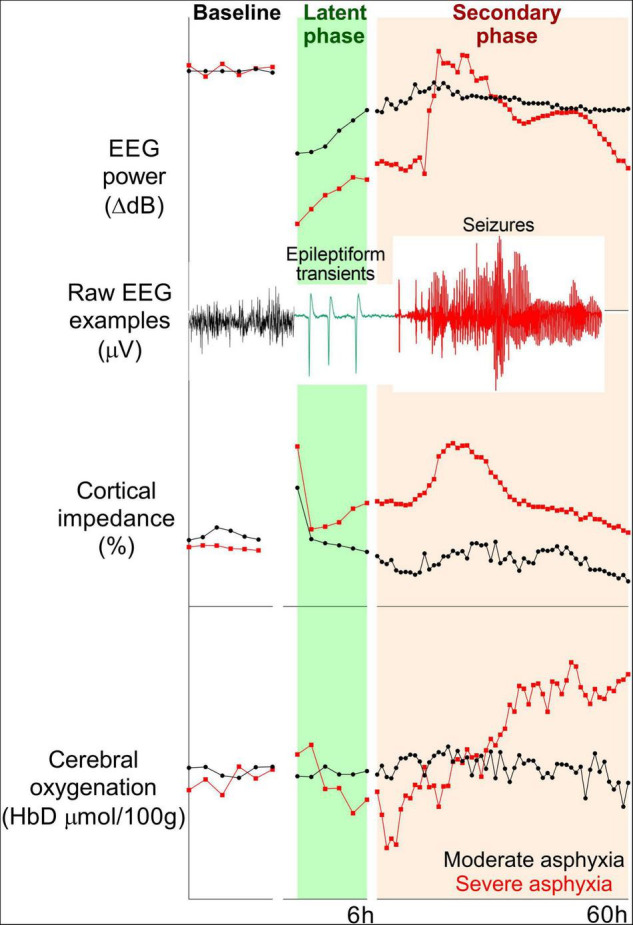
Examples of changes in EEG power (top panel), cortical impedance (middle panel), and cerebral oxygenation (difference in oxygenated and deoxygenated hemoglobin, bottom panel) during baseline and 60 h post-HI recovery in near-term (0.85 gestation) fetal sheep subjected to 15 min (*n* = 2) or 18 min (*n* = 2) complete umbilical cord occlusion. Fifteen and eighteen minutes of asphyxia reflect moderate and severe HI, respectively. In the moderate group, total EEG power remained suppressed, but epileptiform transient activity on a suppressed background was seen during the latent phase. High amplitude, stereotypic seizures developed during the secondary phase. EEG power in the severe group increased with the onset of status epilepticus and subsequently fell below the moderate group. A secondary rise in impedance (delayed cell swelling) was only seen in the severe group. Cerebral oxygenation remained stable in the moderate group, but the severe group had early reduction and subsequent increase in cerebral oxygenation during the secondary phase.

During HI, anoxic depolarization leads to profound suppression of EEG activity. After reperfusion, and recovery of oxidative metabolism at the start of the latent phase, EEG activity is initially highly suppressed, even after milder hypoxia-ischemia. For example, in preterm fetal sheep, the severity of EEG suppression in the first 3 h did not discriminate between mild or severe HI (14). At least in part, this reflects that early EEG suppression is related to a combination of neural dysfunction and endogenous neuro-inhibition mediated by factors such as neurosteroids and sympathetic nervous system activation (15). By contrast, subsequent more rapid recovery of EEG activity reflected less severe injury both in preterm and near-term fetal sheep (e.g., see Figure 1) (14, 16).

Consistent with this, in term infants with moderate-severe HIE, significant suppression of EEG voltage was seen on recordings within 6 h of birth. Rapid recovery of severely suppressed (isoelectric) EEG was associated with normal neurodevelopmental outcomes (17). The predictive value of aEEG changes during the first 6 h of life is relatively limited, although quantitative EEG features may have utility (7). In preterm fetal sheep exposed to moderate-severe HI, abnormal epileptiform activity (spikes, sharp, and slow waves) with suppressed background was reported throughout the latent phase. The peak epileptiform activity was seen around 3–4 h post-HI, and the frequency of sharp waves was associated with greater subcortical neuronal loss (18, 19). This pattern is very different from the typical overall suppression seen in moderate-severe HIE at term (20). However, interestingly a small cohort study in term infants with mild HIE found that early EEG recordings (3–6 h after birth) showed disruption of the sleep cycle, excessive sharp and slow waves and changes in spectral measures in the low-frequency bands compared to healthy term infants (21). We speculate that these maturation and severity related differences may reflect relative sparing of the cortex in this setting. Further studies are needed to assess if these qualitative and quantitative features of EEG can improve early identification of preterm or term infants who would benefit from therapeutic hypothermia.

By contrast with the consistent early suppression of EEG activity after moderate to severe HI, cerebral perfusion typically recovers to control values followed by a delayed (secondary) fall, across multiple species and paradigms. Broadly, the more severe the period of HI the earlier this secondary hypoperfusion occurs and the longer it lasts, with relatively little effect on the depth of the fall (22). This post-ischemic fall in cerebral blood flow is not related to hypotension but rather to increased vascular resistance actively mediated by the sympathetic nervous system (18). For example, in 0.8 gestation fetal sheep subjected to 10 min of HI, delayed cerebral hypoperfusion was associated with EEG suppression, reduced cortical heat production but increased oxygenation, suggesting preserved coupling of blood flow and metabolism (23). Consistent with this, even 30 min of ischemia at the same gestational age was associated with no change in arterio-venous brain oxygen extraction during secondary hypoperfusion, showing that cerebral metabolism was suppressed proportionately to the fall in perfusion (24).

**FIGURE 2:**
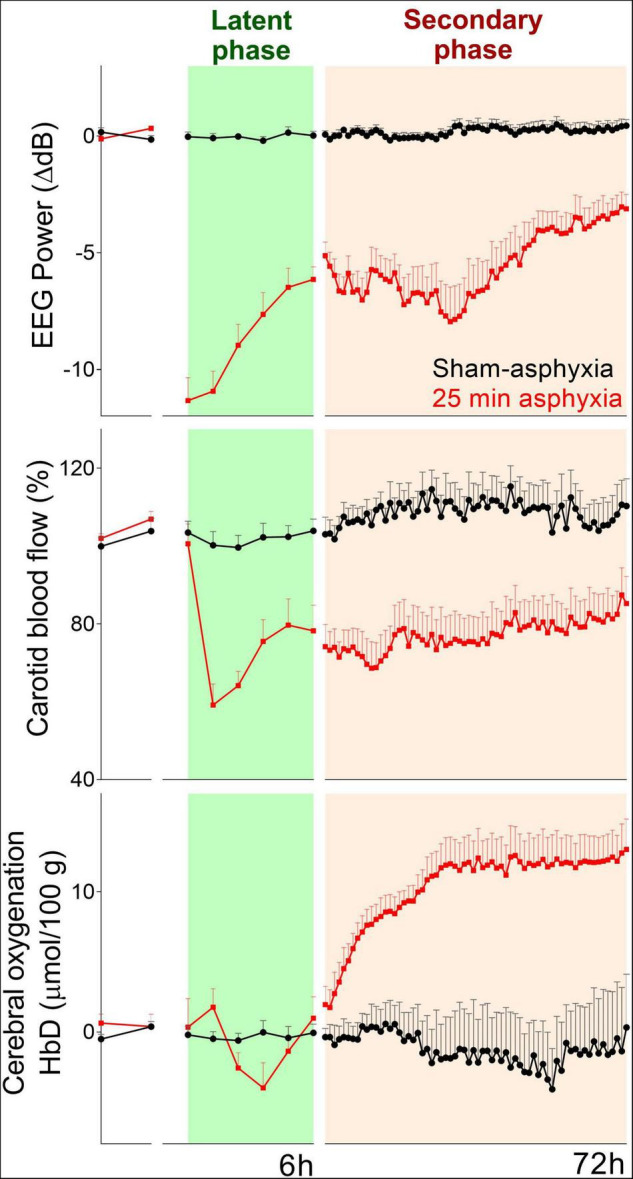
Changes in EEG power (top panel), carotid blood flow (index of cerebral perfusion) (middle panel), and cerebral oxygenation (bottom panel) during baseline and 72 h of post-HI recovery in preterm (0.7 gestation) fetal sheep subjected to sham occlusion (*n* = 4) or 25 min of asphyxia (*n* = 8). Hypoperfusion during the latent phase was associated with a transient decrease in cerebral oxygenation. Despite the sustained reduction in cerebral blood flow, there was an increase in cerebral oxygenation during the secondary phase.

In preterm fetal sheep, however, there was a significant fall in cerebral oxygenation measured by near-infrared spectroscopy (NIRS) at 2–3 h after umbilical cord occlusion, corresponding with the peak of epileptiform transient activity, suggesting a mismatch between perfusion and metabolism (Figure 2). Speculatively, this transient period of secondary hypoxia could exacerbate neural injury (18), although suppressing the epileptiform activity with dizocilpine, a potent anti-excitotoxic agent, was associated with only a minimal improvement in neuronal loss in the cornu ammonis 1/2 region of the hippocampus (25). Clinically, there are limited data on neurophysiological changes during this critical early period after birth. In the pre-hypothermia era, severe HIE in term infants was associated with reduced cerebral blood volume, oxygenation, and cytochrome oxidase during the first 12 h after birth (26).

### Secondary Deterioration: Delayed Mitochondrial Failure and Cerebral Hyperperfusion

Neuropathological processes triggered during the latent phase lead to delayed, “secondary,” deterioration of oxidative metabolism due to failure of mitochondrial function, with cytotoxic edema, seizures and ultimately cell death (6). Clinically, magnetic resonance spectroscopy (MRS) of term infants exposed to birth asphyxia shows a delayed fall in the cerebral concentration of high energy phosphates on the second day after birth. In turn, the severity of impaired oxidative metabolism is associated with greater risk of adverse neurodevelopmental outcomes and death (27, 28).

In near-term fetal sheep, hypoperfusion resolves in the secondary phase, and is followed by a progressive increase in cerebral blood flow peaking at a broadly similar time to cytotoxic edema (29). It is important to note that this is a true hyperperfusion at a time when oxygen consumption is failing, and so leads to increased cerebral oxygenation. The delayed post-ischemia vasodilation in near-term fetal sheep is in part mediated by nitric oxide, and nitric oxide blockade increased subsequent neuronal loss (30). This raises the possibility that secondary hyperperfusion may be protective, perhaps by increasing removal of toxic products. It is interesting to note that in preterm fetal sheep subjected to 25 min of asphyxia, although cerebral blood flow does not increase during the secondary phase, nevertheless, cerebral oxygenation measured by NIRS increases substantially (18). Thus, just as at term, this delayed increase in cerebral oxygenation likely reflects reduced utilization associated with mitochondrial dysfunction. Not surprisingly, therapeutic hypothermia, which reduces brain metabolism, has considerable impact on perfusion. In fetal sheep, therapeutic hypothermia reduced cerebral blood flow in association with an increase in cerebral vascular resistance, with a concomitant suppression of EEG power consistent with reduced brain metabolism (31).

Clinical studies have reported a similar pattern of changes in cerebral perfusion and oxygenation and have assessed the predictive value of these parameters. For example, a reduced cerebrovascular resistive index on transcranial Doppler ultrasound in term infants with moderate to severe HIE, is both consistent with the evidence above for increased perfusion, and is associated with adverse neurodevelopmental outcomes (32). Interestingly, hypothermia markedly attenuates the predictive power of this parameter (33).

Although changes in the secondary phase of evolution are too late to help guide the decision to initiate therapeutic hypothermia, biomarkers in this phase may still help to identify infants who might benefit from additive treatments with hypothermia. Cerebral blood flow and oxygenation measured by NIRS are increased on the first day of life in neonates with moderate-severe HIE, similarly to the studies in fetal sheep discussed above. Importantly, the degree of hyperperfusion was associated with the severity of injury and subsequently increased risk of adverse neurodevelopmental outcomes (34, 35). An increase in cerebral oxygen saturation during hypothermia and rewarming was also associated with injury severity (36). However, variable predictive ability was seen in these small observational studies (37). Moreover, in most of the studies, cerebral oxygen saturation and adverse neurodevelopment were not significantly associated before 24 h of age; speculatively, this may reflect variability in the onset of perinatal injury before birth.

Importantly, several studies have reported a trend for increasing cerebral oxygenation during hypothermia and rewarming to be associated with adverse neurodevelopmental outcomes, compared to stable oxygenation values in neonates with normal MRI findings (36, 38). This suggests that continuous monitoring with NIRS may still offer useful predictive value despite high inter-individual variability. Further, combined assessment of NIRS monitoring with continuous EEG background and seizure recordings may improve the predictive value of cerebral oxygenation (39).

Therapeutic cooling in neonates with moderate-severe HIE can be achieved with either selective head cooling or whole-body cooling. In practice, both these modes result in similar reduction in mortality and major disability (40). Small cohort studies in infants who received whole-body or selective head cooling found that cerebral hemoglobin oxygen saturation was higher in infants with adverse neurodevelopmental outcomes (35, 41), presumptively reflecting reduced oxygen consumption due to greater brain injury. However, there are no clinical data comparing cerebral metabolic alterations with two modes of cooling. A study in newborn piglets showed that selective head cooling with constant rectal temperature was associated with a temperature gradient between cortex and deep brain structures, whereas systemic hypothermia resulted in a more homogenous cooling (42). However, despite the difference in temperature gradient both modes of cooling were associated with a similar reduction in cerebral perfusion and oxygen uptake, indicating a comparable metabolic suppression.

Finally, there is growing recognition that mild HIE can be associated with a significant risk of neurodevelopmental abnormalities. For example, a small cohort study of term infants with mild HIE showed that 11 of 18 had abnormal Doppler findings on the first day of life and that this was associated with white matter abnormalities on MRI (43). Further studies are needed to understand the cerebral metabolism and oxygenation changes after mild HI.

### Postnatal Cerebral Hypoxia in Preterm Infants

Cardiorespiratory instability in preterm infants, related to immature lungs, periodic breathing and apnea and patent ductus arteriosus (PDA), is highly associated with both intermittent and sustained falls in cerebral oxygenation (44, 45). In extremely preterm infants, low cerebral oxygenation saturation during the first 96 h after birth is associated with increased risk of mortality and brain injury, including intraventricular hemorrhage and periventricular leukomalacia (46, 47). Observational studies in very preterm infants have also reported that a higher burden of cerebral hypoxia during the first 72 h, and lower area under the curve for cerebral oxygenation saturation during the first two weeks of life was associated with neurodevelopmental impairment (48, 49). These findings suggest that cerebral oxygenation monitoring might improve outcomes in the early neurocritical care of preterm infants. The phase II SafeboosC trial showed that NIRS monitoring between 3 and 72 h after birth and guideline-based treatments for blood pressure management reduced the duration of cerebral hypoxia in extremely preterm infants (50), but was not associated with improved neurodevelopmental outcomes (51). Phase III trials are now underway to examine if targeting cerebral oxygenation during the immediate transition after birth and whether a NIRS monitoring based approach to improve cerebral oxygenation in extremely preterm infants could reduce neural injury and adverse neurodevelopmental outcomes (52).

### Altered Neurovascular Coupling

In addition to the overall changes in cerebral oxygenation during the secondary phase, there is evidence from assessment of dynamic perfusion with neural activity that cerebrovascular regulation may be impaired (53). A small prospective study of wavelet-based neurovascular coupling in term infants with HIE showed that coherence of aEEG and cerebral oxygen saturation during the first 24 h after birth was higher in infants with normal MRI than those with abnormal MRI findings (54). With a cut-off of a 10% reduction, neurovascular coupling had an area under the curve of 0.808, with a positive predictive value of 94% and negative predictive value of 52% for predicting brain abnormality on MRI, which was better than the total Sarnat score. Interestingly, in an exploratory analysis, infants with mild HIE who had an abnormal MRI also had lower neurovascular coupling than those with a normal MRI. Larger cohorts will be needed to assess the feasibility of neurovascular coupling as a biomarker of injury in the latent phase and to help stratify the severity of injury.

### Impaired Cerebral Autoregulation

Cerebral autoregulation is a physiological mechanism that maintains relatively stable cerebral blood flow during changes in blood pressure. Multiple studies in term infants with moderate-severe HIE have reported impaired cerebral autoregulation during and after therapeutic hypothermia (55, 56). Recent studies have refined time and frequency domain analysis of NIRS parameters to quantify autoregulatory disturbances, such as spectral coherence analysis of mean arterial pressure (MAP) and NIRS parameters (hemoglobin difference) to calculate pressure passivity, moving correlation coefficient between MAP and relative total tissue hemoglobin concentration to calculate time spent below optimal MAP (range of MAP with the most dynamic vascular reactivity), and wavelet-coherence analysis of MAP and cerebral oxygen saturation (55, 57, 58). These studies showed that the extent of autoregulatory disturbances during hypothermia and rewarming are associated with injury severity and adverse neurodevelopmental outcomes. It is important to note that measures during normothermia after rewarming were more predictive of adverse outcomes, highlighting again the impact of hypothermia on brain activity, and the potential value of neuromonitoring after rewarming (56).

A study in a small cohort (*n* = 14) of term infants with mild HIE using multichannel NIRS showed that although overall autoregulation remained normal, there were regional differences in autoregulation that were not associated with MRI abnormalities (59). It is unknown if the regional autoregulation differences are associated with injury patterns in severe HIE. Impaired autoregulation is likely a consequence of vascular dysfunction associated with the ongoing injurious processes. Still repeated hypo- and hyperperfusion due to loss of autoregulation can also lead to further neural injury. A better understanding of these pathological processes is necessary to establish if improving blood pressure stabilization after hypothermia would have an additional neuroprotective effect. In term infants with moderate-severe HIE, low blood pressure variability from 18 to 27 h after birth may be additive with perinatal factors for predicting an adverse EEG profile at 48 h (60), but the long-term predictive value is unclear.

### Seizure-Induced Secondary Metabolic Challenges

The early postnatal period of deranged metabolism and impaired cerebral vascular function in the secondary phase also coincides with peak seizure activity. HIE-associated seizures have typical onset and maximum seizure burden within 24 h after birth (61). Therapeutic hypothermia reduces seizure burden, but nearly half of the cooled babies still have seizures, and the odds of seizures during rewarming are higher (62). The incidence of seizures in preterm infants is much higher than term infants (63). Several clinical studies have shown that seizure burden is a prognostic marker for injury severity and higher risk of mortality and adverse neurodevelopmental outcomes in both preterm and term infants (63, 64). However, these analyses are retrospective and cannot establish if seizures independently contribute to the exacerbation of neural injury or if seizure burden is only a reflection of the severity of the evolving injury.

Understanding the seizure-induced metabolic demand and associated perfusion changes is vital to determine whether seizure-related metabolic disturbances worsen neural injury. For example, a study in near-term fetal sheep subjected to 10 min of asphyxia induced by complete umbilical cord occlusion showed that short duration post-HI seizures of up to 3.5 min were not associated with a fall in cerebral oxygenation. Seizures longer than 3.5 min were associated with a transient mild fall in cerebral oxygenation, which plateaued with a delayed increase in cerebral blood flow (65). Similarly, simultaneous recording of EEG and NIRS in near term infants with HIE showed that during seizures, there is an increase in cerebral metabolic demand and a biphasic hemodynamic response with an initial reduction in oxygenation and perfusion followed by an increase in blood flow (66). Notably, the initial decrease in oxygenation was transient and did not change with the duration of the seizure. The mechanisms mediating the early hemodynamic response are not clearly understood. Collectively, these data suggest that mild oxygenation changes during discrete single post-HI seizures are unlikely to contribute to neural injury. By contrast, in neonatal piglets, post-HI seizures were associated with altered cerebral metabolites on MRS at 24 h and worse neural injury at 72 h; however, causality was not established (67). Furthermore, case studies using simultaneous video EEG recording and diffuse optical tomography in neonates with HIE showed differential spatial distribution of hemodynamic changes across the cortex, likely indicating regional changes in neural activity and perfusion during seizure propagation (68).

While we know that seizures are more common in preterm than term-born neonates, little is known about the changes in cerebral metabolism and perfusion during seizures in the preterm brain. A study in 12 preterm infants showed that seizures are associated with pressure passive fluctuations in cerebral blood flow velocity that could increase the risk of intracerebral hemorrhage (69). Understanding the mechanisms that regulate perfusion during neonatal seizures and the contribution of seizure-induced metabolic changes to neural injury is important for improving seizure management.

In near-term fetal sheep, seizure activity during the secondary phase is also associated with increased excitotoxic index in the brain (70), likely due to failure of reuptake. Moreover, the phase of secondary deterioration is also associated with upregulation of neuronal injury markers such as Tau protein, neuron-specific enolase, S100 calcium-binding protein beta and neurofilament light chain protein, inflammatory proteins such as glial fibrillary acidic protein, interleukins (IL-6, IL-8, and IL-10), TNF-alpha and oxidative stress markers in the plasma (7).

### Long-Term Neurophysiological Alterations

The secondary phase after HI resolves after 3–4 days into a tertiary phase involving persistent inflammation, delayed evolution of cystic injury and repair and reorganization processes (12, 13, 71). On the one hand these processes are essential for reorganizing the brain, but the very prolonged exposure to inflammation raises the intriguing possibility that there could be an extended therapeutic window of opportunity to improve recovery.

Studies of neurophysiological recovery in fetal sheep after HI showed persistent suppression of overall EEG power during the tertiary phase, with altered maturational changes in spectral edge frequency distribution associated with sleep-state development and impaired or absent recovery of sleep-state activity at term (72, 73). Similarly, clinical data show that the neurophysiological and metabolic disturbances after HI persist for weeks. The time of onset and quality of sleep-wakefulness in neonates with HIE is associated with the severity of the injury and impaired neurodevelopment (74). Small prospective and retrospective studies have reported that functional integrity of the ascending pathways measured by somatosensory evoked potential was altered in term infants with HIE after rewarming and during the first 2 weeks of life, and these alterations were predictive of MRI abnormalities (75, 76).

Magnetic resonance spectroscopy studies in term infants with moderate-severe HIE have shown that the early increase in lactate concentrations during the secondary phase resolves, but low concentrations of NAA in the deep gray matter structures during 5–14 days after birth were associated with adverse neurodevelopmental outcomes at 2 years of age (77, 78). It is important to note that while these functional measures at single time points are associated with adverse outcomes, long-term studies with continuous or repeated assessments will be required to identify neurophysiological features associated with the delayed evolution of injury to guide the development of future therapeutic strategies.

### Iatrogenic Changes in Cerebral Oxygenation

Term infants with HIE and preterm infants are exposed to various regimens for clinical management and for trials examining neuroprotective treatments. A better understanding of metabolic and vascular effects of iatrogenic factors in these babies may offer therapeutic opportunities. Treatments that alter cerebral metabolism and/or perfusion during the early post-HI recovery may further exacerbate the neural injury. For example, in preterm fetal sheep, post-HI treatment with dexamethasone was associated with cerebral deoxygenation during the latent phase, increased abnormal ictal activity during the secondary phase and greater subcortical neuronal loss (79, 80). Similarly, delayed treatment with high-dose (5,000 IU) intravenous boluses of human recombinant erythropoietin starting at 6 h after moderate-severe HI in preterm fetal sheep was associated with increased cerebral vascular resistance and prolonged cerebral hypoperfusion without a corresponding reduction in EEG power (81). This sustained mismatch between perfusion and brain activity during the secondary phase was associated with the development of cystic white matter injury.

Postnatal cardiorespiratory instability has cumulative effects on cerebral oxygenation in preterm infants. In addition, iatrogenic factors can also contribute to cerebral hypoxia. Hemodynamic management of preterm infants is challenging, as the definition of the lower limit of blood pressure in extremely preterm infants is controversial and may promote overtreatment. Importantly, after severe asphyxia in near-term fetal lambs infusion of the inotropic agent, dopamine was associated with only a transient improvement in arterial blood pressure, and did not prevent terminal hypotension (82). Clinically, nearly 30% of extremely preterm infants receive inotropes for treatment of hypotension [defined as mean BP (mmHg) lower than infant’s gestational age] on the first day of life but there is little evidence for benefit (83). In a prospective blinded study in extremely preterm infants, dopamine improved blood pressure but did not prevent cerebral hypoxia (84).

Patent ductus arteriosus is a common cardiovascular complication in preterm infants. Indomethacin is effective in closing PDAs; however, an RCT in preterm infants showed that a high dose of indomethacin was associated with a significant reduction in cerebral blood flow and oxygen delivery (85). This is of concern since, despite conflicting evidence, prophylactic indomethacin is administered inconsistently but relatively commonly to extremely preterm infants to promote PDA closure and ultimately to improve cerebral perfusion (86). Further, an observational study in very preterm infants showed that prophylactic indomethacin treatment was associated with a mild but significant increase in cerebral oxygen extraction, denoting reduced cerebral blood flow (87). These findings suggest that further examination of the cerebrovascular impact of prophylactic indomethacin treatment is warranted.

Pharmacological interventions are routinely used for sedation and analgesia in neonates undergoing therapeutic hypothermia to reduce stress, but management of sedation is not standardized (88), and there are very limited clinical data of the impact of commonly used sedatives on brain injury in term neonates. Secondary analysis of the Magnetic Resonance Biomarkers in Neonatal Encephalopathy study suggested that pre-emptive morphine sedation during therapeutic hypothermia was not associated with improvement in neurodevelopment, and that neonates who received morphine were more likely to develop hypotension and had longer hospital stays (89). It is also important to consider the clearance of many agents is reduced during hypothermia and so metabolite accumulation can occur over time (89). A small retrospective study reported that there was no relationship between the cumulative dose of fentanyl during therapeutic hypothermia and neurodevelopmental outcomes; however, further pharmacokinetic studies are needed (90). Despite limited data on superiority over opiates, the selective alpha-1 adrenergic receptor dexmedetomidine is increasingly being used as a sedative in critically ill term and preterm infants (88). Worryingly, in a prospective study, dexmedetomidine administration was associated with greater instability of oxygenation and increased need for respiratory support (91). Moreover, in newborn piglets, combined treatment with dexmedetomidine and therapeutic hypothermia after HI reduced clearance of dexmedetomidine, leading to increased plasma concentrations that were associated with greater risk of cardiac arrest and neuronal loss (92). Randomized controlled trials of sedatives during therapeutic hypothermia are needed to establish their safety and long-term effects.

## Conclusion

Small prospective and retrospective studies have shown that the overall higher cerebral oxygenation, altered neurovascular coupling and impaired cerebral autoregulation during and after hypothermia are associated with adverse neurodevelopmental outcomes. Large, well-designed, prospective studies are now required to assess the viability of these measures as a cot-side tool. Excitingly, early data from small prospective studies have shown that measures such as neurovascular coupling may be valid prognostic biomarkers for mild HIE; validation studies in larger cohorts are needed. The majority of the neuromonitoring data in present studies have been acquired during or after hypothermia. Future studies examining the dynamic measures of cerebrovascular function early after birth in both term and preterm infants are required to assess them as markers for early identification of at-risk infants. Finally, data on the potential for adverse iatrogenic effects on cerebral oxygenation suggest the need for careful assessment of acute and long-term neural effects of clinical management in critically ill neonates and implementing evidence-based changes in practice. Large clinical trials with comparative approaches are required to standardize data collection and analysis for cerebral oxygen measurements, and to establish true normative values in preterm infants.

## Author Contributions

SD, LB, and AG conceptualized the review. SD wrote the first draft of the manuscript. All authors critically revised the manuscript and approved the final manuscript as submitted.

## Conflict of Interest

The authors declare that the research was conducted in the absence of any commercial or financial relationships that could be construed as a potential conflict of interest.

## Publisher’s Note

All claims expressed in this article are solely those of the authors and do not necessarily represent those of their affiliated organizations, or those of the publisher, the editors and the reviewers. Any product that may be evaluated in this article, or claim that may be made by its manufacturer, is not guaranteed or endorsed by the publisher.
